# Effects of ozone therapy applied topically, by bagging, or both on the healing of clean wounds induced in rat’s skin

**DOI:** 10.1590/acb397024

**Published:** 2024-10-07

**Authors:** Bárbara Di Martino Frezza, Sheila Canevese Rahal, Ivan Felismino Charas dos Santos, Bruna Martins da Silva, José Ivaldo de Siqueira Silva, Gustavo Manea Ferreira, Michel de Campos Vettorato, Jéssica Leite Fogaça, Miriam Harumi Tsunemi, Carlos Eduardo Fonseca-Alves, Claudia Helena Pellizzon, Vinicius dos Santos Rosa

**Affiliations:** 1 Universidade Estadual Paulista – School of Veterinary Medicine and Animal Science – Botucatu (SP) – Brazil.; 2 Universidade Estadual Paulista – School of Veterinary Medicine and Animal Science – Department of Surgery and Animal Reproduction – Botucatu (SP) – Brazil.; 3 Universidade Federal de Rondônia – Department of Veterinary Medicine – Rolim de Moura (RO) – Brazil.; 4 Universidade Cruzeiro do Sul – Department of Veterinary Medicine – São Paulo (SP) – Brazil.; 5 Centro Universitário do Vale do Ipojuca – Caruaru (PE) – Brazil.; 6 Colégio Cívico Militar, Técnico em Radiologia – Araçatuba (SP) – Brazil.; 7 Faculdades de Tecnologia do Estado de São Paulo – Botucatu – São Paulo (SP), Brazil.; 8 Universidade Estadual Paulista – Department of Biostatistics – Institute of Biosciences – Botucatu (SP) – Brazil.; 9 Universidade Estadual Paulista – Institute of Bioscience – Department of Structural and Functional Biology – Botucatu (SP) – Brazil.; 10 Universidade do Oeste Paulista – Department of Veterinary Medicine – Presidente Prudente (SP) – Brazil.

**Keywords:** Ozone, Sunflower Oil, Wound Healing, Tensile Strength

## Abstract

**Purpose:**

This study aimed to evaluate the effects of ozone therapy applied topically and/or by bagging on the healing of clean wounds induced in rat’s skin.

**Methods:**

One hundred and twenty male rats of about 16 weeks old was divided into five groups: G1) saline solution (0.9%); G2) sunflower oil; G3) ozonated sunflower oil; G4) ozone bagging; G5) association of ozonated sunflower oil and ozone bagging. The wounds were evaluated through macroscopic, morphometric, histopathologic, and tensile strength analyses.

**Results:**

Analysis among groups showed a lower percentage of wound contraction in G1 compared to G4 only in M_7D_. The tensile strength of the wounds showed differences among groups in the seventh (M_7D_) and the 14^th^ (M_14D_) postoperative day, and among time points in G1 (M_14D_ > M_7D_). The elongation of the wounds showed differences in G3 (M_7D_ > M_14D_). Histological evaluation of the wounds showed significant change in bleeding, mixed to mononuclear infiltrate, congestion, and tissue disorganization for tissue organization between groups and time points.

**Conclusions:**

Ozone therapy applied topically and/or by bagging was not deleterious to the healing of clean wounds induced in rat’s skin, but ozone bagging showed the best contribution to the healing process.

## Introduction

Ozone therapy can activate the natural healing capacity if used in small and safe ozone doses^1^. Some biological responses related to ozone include mild activation of the immune system and enhancement of the release of growth factors, induction of antioxidant enzymes, improvement of blood circulation and oxygen delivery to ischemic tissue, activator of stem cells, among other responses^2^^,^ 3.

Due to the versatility of ozone, several forms of administration have been described, such as topical, subcutaneous, intramuscular, intradiscal, intracavitary, intravaginal, intraurethral, vesical, and ozonized hemotherapy, among others^2^^,^ 4. However, ozone is a toxic gas that should not be breathed and requires caution in its use^3^.

The ozone therapy is non invasive and low cost and has been used in several clinical applications, such as infected cutaneous wounds, bed sores, necrotizing fasciitis, chronic ulcers, and initial gangrene^3^^,^ 5 ^–^ 8. Topical therapy with ozonated oil or ozonated solutions seems to enhance wound healing due to cleansing effects, antimicrobial properties, release of oxygen in hypoxic tissues, and the stimulation of fibroblasts^2^^,^ 9. Parallelly, sunflower oil is considered adequate to carry ozone and has a longer shelf life, but the peroxidation degree must be controlled since it can affect the efficacy^8^^,^ 10. On the other hand, olive oil, sesame oil, and many other unsaturated fatty acids can be used^10^.

Ozone bagging therapy is another modality in which a plastic bag is placed around the wound, and the ozone/oxygen mixture is transported into the bag^6^.

Since the type of topical therapy may influence the ozone efficacy, this study aimed to evaluate the effects of ozone therapy applied topically and/or by bagging on the healing of clean wounds induced in rat’s skin. The hypothesis was that ozonated sunflower oil associated with ozone bagging could be more effective to promote healing of clean wounds in rats, compared with the same therapies administered isolated.

## Methods

### Animals and experimental design

This study was approved by the Ethics Committee for Animal Care and Use of the School of Veterinary Medicine and Animal Science, Universidade Estadual Paulista “Júlio de Mesquita Filho”, in Botucatu, SP, Brazil (no. 1274-2018).

One hundred and twenty Wistar rats, intact males, of about 16 weeks old and weighing from 350 to 450 g were used. The rats were housed in a room at 22–25ºC temperature, with 40–60% relative humidity, on a 12-hour light/dark cycle. Each rat was kept in an individual plastic cage, which was cleaned every 48 hours with water and neutral soap, with food and water *ad libitum*. A period of 15 days was established for adaptation to the environment. The rats were randomly allocated into five groups (n = 24 per group), using Research Randomizer program (Version 4.0), based on wound treatment protocols:

- G1: saline solution (0.9%);
- G2: sunflower oil;
- G3: ozonated sunflower oil;
- G4: ozone bagging;
- G5: association of ozonated sunflower oil and ozone bagging.

Sunflower oil has linoleic acid (48–74%) and oleic acid (14–39%) in its composition, and ozonation was carried out with Trailigaz Labo model 12-02 ozone generator. Before starting the study, samples of sunflower oil and ozonated sunflower oil were sent for fungal and bacterial cultures, which had negative results. The ozone gas used for bagging was obtained using O&L 1.5 RM ozone generator.

### Anesthesia, surgical procedure and treatments

The rats were anesthetized with etamine chloridrate (30 mg/kg) and xylazine (2 mg/kg) administered intraperitoneally (IP). The epilation was performed on the dorsal and thoracic region and disinfected with 2% chlorhexidine solution.

A circular wound (1-cm diameter) was made on the dorsum of each animal by a metallic punch, and a fragment of skin and subcutaneous tissues was removed with the preservation of the dorsal muscle fascia. The wounds of G1, G2 and G3 were treated every 24 hours with 0.015 mL (one drop) of medication, according to the assigned treatment.

The wounds, abdomen, and hind limbs of the animals of G4 were bagged with a plastic bag (40 × 50 cm). The top of the plastic bag was closed by a nylon tie-rap and bandaging tape in axillary region of the rat. The ozone was introduced through a polyvinyl chloride urinary catheter from the ozone generator (Ozone & Life, O&L 1.5 RM) into the plastic bag. A mixture of ozone and oxygen were pumped into the airtight bag for 5 min (ozone concentration of 30 µg/mL). Then, the ozone generator was turned off, and the bag was maintained in place for a period of 10 min. These wounds were treated every 72 hours.

The wounds of the animals of G5 were treated each 24 hours with 0.015 mL (one drop) of ozonated sunflower oil, and ozone bagging every 72 hours, as used in G4.

Sterile gauze moistened with ozonated saline solution (0.9%) (47 µg/mL) was used for cleaning the wounds before each treatment, and all wounds were left uncovered. All treatments were administered at 7 a.m., and the study was conducted as a double-blinded study. No antibiotics or anti-inflammatories were administered during the study. Tramadol hydrochloride [10 mg/kg, each 12 hours (BID), subcutaneous] was administered for 48 hours to manage postoperative pain. The surgical procedures were conducted by the same experienced person at 7 a.m., following the asepsis protocols.

### Macroscopic and morphometric evaluations

The wounds were assessed every day for signs of infection, presence of crust, and granulation tissue. Photographic records were taken using a digital camera, and a software Image tool (Image^®^) was used to measure skin wound areas on the seventh (M_7D_) and the 14th (M_14D_) postoperative day. The percentage of skin wound contraction was determined as Eq. 1:


$$\text{Wound contraction}=\frac{\text{Initial}\text{wound area-Area on day of the measurement}}{\text{Initial wound area}}\times 100\%$$ (1)

Computed tomography (CT) was used for measure wound depth evaluation of the wounds. For that, a helical CT (Shimadzu SCT-7800CT, Kyoto, Japan) was used to evaluate wound depth on the M_7D_ and M_14D_. After anesthesia with ketamine chloridrate (30 mg/kg) and xylazine (2 mg/kg) (IP), the rats were placed in ventral recumbency, and sequential transverse images of the wound were acquired. The CT scanning parameters were as follows: 120 kVp, 100 mA, 1.0-mm-thick slices, a pitch of 1 s/rotation. After image reconstruction in multiplanar reformation (MPR) with Voxer 3D 6.3 (Barco, Kortrijk, Belgium), the wounds were measured in the axial plane, by using a line drawn from the base of the skin wound to its top in the middle region of the lesion.

### Microscopic evaluation

Twelve rats in each group were submitted to euthanasia with thiopental sodium (120 mg/kg) and lidocaine without vasoconstrictor (7 mg/kg) administered intraperitoneally on the M_7D_ and M_14D_. The wounds of all groups were collected with a 2-cm margin around the lesion, and the depth was including the dorsal muscular fascia to proceed with the histological analysis (n = 5) and the evaluation of the mechanical properties (n = 7).

The specimens for histological evaluation were fixed in 10% neutral-buffered formalin, and, after fixation, the tissue samples were maintained in 70% alcohol, and then embedded in paraffin. Sections of 4–5 μm of thickness were obtained and stained with hematoxylin and eosin (HE), which were evaluated under an optical microscope at 10X and 40X magnification, and documented by photomicrography. Congestion of blood vessels, inflammatory cells infiltrate, and tissue organization were classified semi-quantitatively as follows: low intensity (degree 1), moderate intensity (degree 2), and high intensity (degree 3).

### Mechanical testing of wounds

A standardized rectangular portion of the skin with a central healing wound was fixed by clamps on both sides on the universal testing machine (EMIC, DL10000). The samples were tested in tension until failure, elongation rate, and ultimate elongation. The strength of the wound was defined as the ultimate tensile force.

### Statistical analysis

Statistical analysis was performed using R software (Version 3.4.4). The normality of the data was assessed with Shapiro-Wilk’s test. In each group, a comparison between time points was performed using an analysis of variance (ANOVA) with the time point as a random effect, followed by multiple comparisons between time points. The Mann–Whitney’s test was used to compare the groups. Histological variables were analyzed with the Fisher’s test. Differences were considered statistically significant with *p* < 0.05.

## Results

### Clinical evaluation and macroscopic evaluation

The body temperature was maintained in accordance with reference variation for the species, ranging from 36.2 to 37.1°C. The body mass decreased between the first and second day after wound induction, but increased from the third day onwards. There were no deaths during surgical procedures or the treatment period of the wounds. The rats did not damage the wounds during the follow-up period.

**Figure 1:**
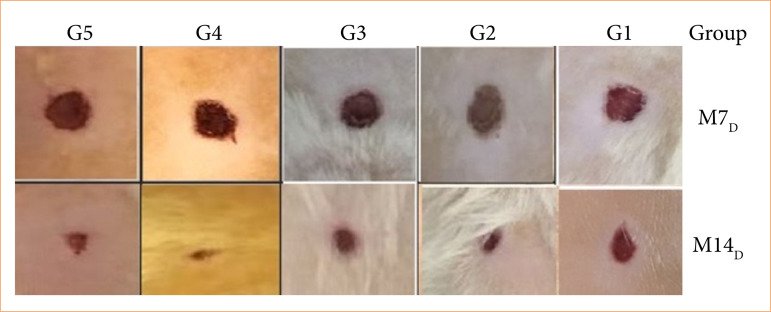
Photographs of circular wounds on the dorsum of rats that were treated with saline solution (G1), sunflower oil (G2), ozonated sunflower oil (G3), ozone bagging (G4), and association of ozonated sunflower oil and ozone bagging (G5) on the 7^th^ (M_7D_) and the 14^th^ (M_14D_) postoperative day.

Source: Elaborated by the authors.

In all the wounds, no signs of infection were observed during the treatment period (Fig. 1). Crust and granulation tissue were seen on the M_7D_ and M_14D_, respectively.

### Wound measurements

In all groups, significant differences (*p* = 0.0002) in the wound areas were observed between M_7D_ and M_14D_ (Table 1).

**Table 1:** Areas (cm^2^) of wounds induced in rats assessed on the seventh (M_7D_) and 14^th^ (M_14D_) postoperative day treated with saline solution (G1), sunflower oil (G2), ozonated sunflower oil (G3), ozone bagging (G4), association of ozonated sunflower oil and ozone bagging (G5)*.

Groups	M_7D_ (Mean ± standard deviation)	M_14D_ (Mean ± standard deviation)
G1	4.9 ± 1.1^Aa^	1.5 ± 0.7^Ba^
G2	3.18 ± 0.8^Aa^	1.13 ± 0.39^Ba^
G3	3.22 ± 1.3^Aa^	1.29 ± 1.05^Ba^
G4	4.18 ± 1.53^Aa^	1.04 ± 0.5^Ba^
G5	3.86 ± 2.01^Aa^	0.92 ± 0.25^Ba^

* Means followed by different capital letters in the same line were significantly different by Mann–Whitney’s test (*p* < 0.05).

Means followed by different small letters in the same column were significantly different by analysis of variance test (*p* < 0.05). Source: Elaborated by the authors.

Concerning the percentage of wound contractions, a significant increase (*p* = 0.0002) was identified in all groups between both time points. A significant difference (*p* = 0.0319) was observed in the contraction values between G1 and G4 (G4 > G1) on the M_7D_ (Table 2).

**Table 2:** Percentage of contraction of wounds induced in rats assessed on the seventh (M_7D_) and 14^th^ (M_14D_) postoperative day treated with saline solution (G1), sunflower oil (G2), ozonated sunflower oil (G3), ozone bagging (G4), association of ozonated sunflower oil and ozone bagging (G5)*.

Groups	M_7D_ (Mean ± standard deviation)	M_14D_ (Mean ± standard deviation)
G1	41.59 ± 8.7^Aa^	82 ± 8.6^Ba^
G2	63.33 ± 12.98^Aab^	86.71 ± 6.12^Ba^
G3	62.058 ± 15.2^Aab^	83.68 ± 14.55^Ba^
G4	56.87 ± 19.37^Ab^	89.32 ± 5.12^Ba^
G5	55.88 ± 12.5^Aab^	89.88 ± 2.74^Ba^

* Means followed by different capital letters in the same line were significantly different by Mann–Whitney’s test (*p* < 0.05).

Means followed by different small letters in the same column were significantly different by analysis of variance test (*p* < 0.05). Source: Elaborated by the authors.

The values of depth of the wound showed a significant decrease between M_7D_ and M_14D_ in G1 (*p* = 0.0000), G2 (*p* = 0.0368) and G5 (*p* = 0.0133) (Table 3).

**Table 3:** Values of depth (cm) of skin wounds induced in rats assessed on the seventh (M_7D_) and 14^th^ (M_14D_) postoperative day treated with saline solution (G1), sunflower oil (G2), ozonated sunflower oil (G3), ozone bagging (G4), association of ozonated sunflower oil and ozone bagging (G5).

Groups	M_7D_ (Mean ± standard deviation)	M_14D_ (Mean ± standard deviation)
G1	0.62 ± 0.23^Aa^	0.58 ± 0.22^Ba^
G2	0.74 ± 0,11^Aa^	0.58 ± 0.2^Ba^
G3	1,0 ± 0.35^Aa^	0.72 ± 1.8^Aa^
G4	0.93 ± 0.25^Aa^	0.65 ± 0.19^Aa^
G5	0.81 ± 0.09^Aa^	0.62 ± 0.15^Ba^

*Means followed by different capital letters in the same line were significantly different by Mann–Whitney’s test (*p* < 0.05). Means followed by different small letters in the same column were significantly different by analysis of variance test (*p* < 0.05). Source: Elaborated by the authors.

### Mechanical testing of wounds

The intergroups evaluations showed a significant difference (*p* = 0.00) in the tensile force values between G1 and G2/G3 (G3 > G1; G2 > G1) at M_7D_, and at M_14D_ significant variations (*p* = 0.00) were identified between the following groups: G1 > G4; G3 > G1, G2; G4 > G2; and G5 > G1, G2. On the other hand, the wounds submitted to treatment with saline solution (0,9%) demonstrated a significant increase (*p* = 0.00) in tensile force values between M_7D_ and M_14D_ (Table 4).

The wounds treated with ozonated sunflower oil (G3) showed significant decrease (*p* = 0.040) in elongation values between M_7D_ and M_14D_, and the wounds treated with salina solution (0.9%) demonstrated significant increase (*p* = 0.0207) in ultimate elongation values between both time points (Table 4).

**Table 4:** Tensile force (TF), elongation (ELO), and ultimate elongation (U-ELO) of wounds induced in rats assessed on the seventh (M_7D_) and 14^th^ (M_14D_) postoperative day treated with saline solution (G1), sunflower oil (G2), ozonated sunflower oil (G3), ozone bagging (G4), association of ozonated sunflower oil and ozone bagging (G5).

Groups	M_7D_ (Mean ± standard deviation)	M_14D_ (Mean ± standard deviation)
**(TF) (N)**		
G1	1.6 ± 0.5^Aa^	3.6 ± 1.8^Ba^
G2	2.83 ± 0.95^Aab^	2.03 ± 0.98^Aa^
G3	3.188 ± 1.1^Ab^	4.32 ± 1.62^Ab^
G4	3.35 ± 2.11^Aab^	2.55 ± 0.82^Aab^
G5	7.713 ± 3.68^Aab^	7.86 ± 2.26^Aab^
**ELO (mm)**		
G1	2.0 ± 1.0^Aa^	1.7± 1.2^Aa^
G2	3.04 ± 1.59^Aa^	1.7 ± 1.45^Aa^
G3	3.31 ± 1.35^Aa^	1.91 ± 0.67^Ba^
G4	2.84 ± 1.31^Aa^	3.16 ± 1.31^Aa^
G5	3.30 ± 1.0^Aa^	2.75 ± 1.2^Aa^
**U-ULO (N/mm)**		
G1	1.1 ± 1.0^Aa^	2.7 ± 1.73^Ba^
G2	1.40 ± 1.46^Aa^	1.97 ± 0.93^Aa^
G3	1.30 ± 0.59^Aa^	2.25 ± 0.91^Aa^
G4	1.29 ± 0.73^Aa^	1.73 ± 1.42^Aa^
G5	2.47 ± 1.0^Aa^	1.99 ± 0.85^Aa^

*Means followed by different capital letters in the same line were significantly different by Mann–Whitney’s test (*p* < 0.05). Means followed by different small letters in the same column were significantly different by analysis of variance test (*p* < 0.05). Source: Elaborated by the authors.

### Histological evaluation

Regarding histological evaluation of the wounds (congestion of blood vessels, hemorrhage, inflammatory infiltrate, and tissue organization) among groups on the M_7D_, significant variation in hemorrhage from intense to mild was observed between G1 (saline solution) and G2 (sunflower oil) (*p* = 0.0263) (G2 > G1), as well as significant changes from mixed to mononuclear inflammatory infiltrate between G1 (saline solution) and G4 (ozone bagging) (*p* = 0.0389) (G1 > G4).

On the M_14D_, a significant variation was observed in congestion from mild to moderate between G1 (saline solution) and G3 (ozonated sunflower oil) (*p* = 0.0224) (G3 > G1), in tissue disorganization for tissue organization between G1 (saline solution) and G4 (ozone gas insufflation in bags) (*p* = 0.0117) G1 > G4), and between G2 (sunflower oil) and G4 (ozone bagging) (*p* = 0.0389) (G2 > G4).

In the groups, significant variation (*p* = 0.0256) occurred from ulcerated to non-ulcerated epithelium in wounds treated with ozonated sunflower oil (G3) between both time points, and significant variation from mixed to mononuclear inflammatory infiltrate in wounds treated with saline solution (G1) between M_7D_ and M_14D_ (*p* = 0.0389).

**Figure 2:**
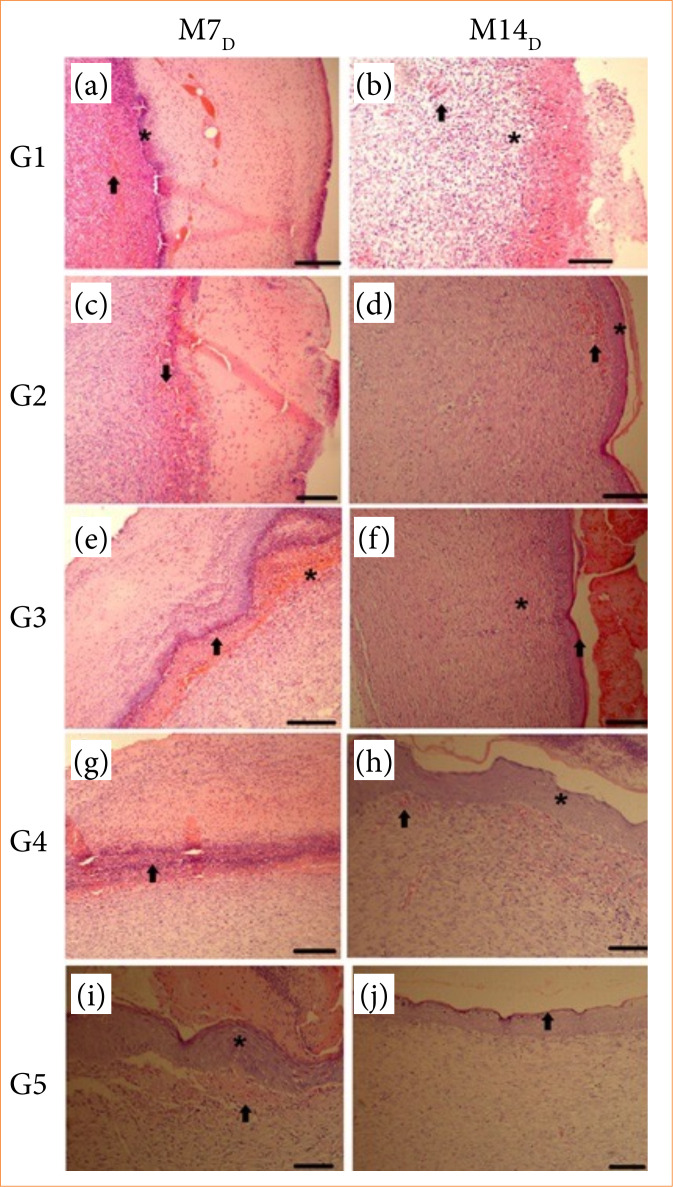
Representative photomicrographs of skin wounds in Wistar rats treated with saline solution (G1), sunflower oil (G2), ozonated sunflower oil (G3), ozone bagging (G4), and association of ozonated sunflower oil and ozone bagging (G5) on the 7^th^ (M_7D_) and the 14^th^ (M_14D_) postoperative day. **(a)** Congestion of blood vessels (arrow), areas with multifocal bleeding, and intense polymorphonuclear inflammatory infiltrate (asterisk). **(b)** Congestion of blood vessels (arrow), inflammation with polymorphonuclear infiltrate (asterisk), and areas of multifocal bleeding. **(c)** Neovascularization and congestion in newly formed vessels (arrow), in addition to intense polymorphonuclear inflammatory infiltrate (asterisk). **(d)** Area of mild hemorrhage (arrow), complete wound healing (asterisk), and absence of inflammation. **(e)** Intense polymorphonuclear inflammatory infiltrate (arrow) and area of moderate bleeding (asterisk). **(f)** Formation of new epithelium beneath the scab (arrow), linear organization of collagen fibers (asterisk), and absence of significant inflammation. **(g)** Intense polymorphonuclear inflammatory infiltrate (arrow). **(h)** Neovascularization (arrow), new epithelium formed beneath the crust (asterisk), and moderate inflammatory infiltrate (triangle). **(i)** Neovascularization (arrow) and new epithelium beneath the crust (asterisk), with moderate inflammatory infiltrate (triangle). **(j)** Complete wound healing (arrow) and absence of inflammation. (hematoxylin and eosin, 40X).

Source: Elaborated by the authors.

Figure 2 represented the histopathological evaluation of the wounds during the treatment period.

## Discussion

The present study aimed to evaluate the effects of ozone therapy applied topically and/or by bagging on the healing of clean wounds induced in rat’s skin, since the ozone therapy could induce positively on wound healing, and the type of topical therapy may influence the ozone efficacy. The hypothesis that ozonated sunflower oil associated with ozone bagging could be more effective in promoting the healing of clean wounds in rats compared with the same isolated therapies was not confirmed in the current study.

The wounds induced in the present study were classified as clean and, through macroscopic analyses, kept its classification as clean throughout the study in all groups. Thus, bactericidal and antifungal effects of ozone^6^^,^ 11 could not be proven *in vivo*. Ozone has been reported to promote a decrease in bacterial colonization due to an inactivation of microorganisms and a release of decomposition products such as reactive oxygen species and peroxides^10^. In addition, ozone applied as ozonated oil, as used in animals of G3, seems to have higher antimicrobial activity than ozonated saline^6^.

The depth of the wounds, area measurement, and the percentage of contraction of the wounds did not show statistical differences among groups on the 14^th^ postoperative day. However, a significant increase in the wound contraction percentage was observed between G1 (saline solution) and G4 (ozone bagging) on the seventh postoperative day. This period is one of the most appropriate to evaluate experimentally induced skin wounds since after the 10^th^ to 12^th^ days the wound area becomes more difficult to accurately delineate^12^. In a study of skin wounds (diameter of 6 mm) induced in guinea pigs, groups treated with ozonated olive oil showed a smaller wound size and residual wound area, respectively, at five and seven days after surgery as compared to treatment with olive oil and the untreated group^13^. Since wound contraction occurs during the proliferative phase, associated with fibroblast migration from wound margins and subsequent differentiation into myofibroblasts^14^^,^ 15, a greater stimulation of these cells by ozone in G4 probably occurred. In addition, the ozone exhibited no cytotoxic effects on fibroblasts and induced fibroblast migration in cell culture assays^16^.

Variable degrees of bleeding were observed in histological evaluation on the seventh postoperative day, but statistical significance from intense to mild was detected only between G1 (saline solution) and G2 (sunflower oil). Since inflammation phase occurs due to fibrin clot formation and platelet degranulation, which releases chemotactic factors^17^, a high degree of bleeding at this stage may contribute to delayed wound healing, with the maintenance of vascular response. In a study that produced incisional wounds in the tail of mice, both ozonated water and ozonated gel had hemostatic capability because bleeding time had been shortened compared to the untreated group^18^. Although mild to moderate hemorrhage scores were found in G4 and G5, in which bagging was used, this could not be seen in G3 (ozonated sunflower oil), that presented mild to intense scores. On the other hand, regarding congestion, there was a significant variation from mild to moderate between G1 (saline solution) and G3 (ozonated sunflower oil) on the 14^th^ postoperative day. In addition, G1 had a mild score in most of the wounds compared to the other groups, showing a good evaluation of this variable.

The inflammatory infiltrate was present on the seventh postoperative day in all groups, but there was a statistical difference from mixed to mononuclear infiltrate only in G1 (saline solution) and G4 (ozone bagging). Also, evaluating between time points, only G1 showed a difference from mixed to mononuclear infiltrate on the 14^th^ postoperative day. The presence of a mixed infiltrate rather than a mononuclear one suggests the persistence of the inflammatory phase in G1 (saline solution). In general, after the initial inflammatory phase, monocytes are converted into macrophages, which are responsible not only for the phagocytosis and digestion of tissue debris and neutrophils, but also for the secretion of growth factors and cytokines, which needed to initiate the proliferative phase^19^^,^ 20.

Tissue organization on the 14^th^ postoperative day showed a statistical difference between G1 (saline solution) and G4 (ozone bagging), as well as between G2 (sunflower oil) and G4, with G4 being better in both cases. The remodeling phase of the wound requires the deposition of organized collagen fibers to obtain a structure as close to normal as possible^19^^,^ 20. The capacity of the ozone for accelerating wound healing has been associated with a stimulation of tissue reconstruction5. The decomposition of ozone in peroxides seems to stimulate tissue repair by improving oxygenation^10^. In addition, hydrogen peroxide acts as a messenger for cytokine induction^21^.

Regarding mechanical testing of the wounds, differences were observed only in tensile strength between G1 (saline solution) and G3 (ozonated sunflower oil), and between G2 (sunflower oil) and G3 (ozonated sunflower oil) at the seventh postoperative day, suggesting that G3 had the highest capacity to withstand loads before failure. In general, the tensile strength of a wound increases progressively in parallel with the growth of collagen agglomeration^19^.

One of the limitations of the present study is the lack of an immunohistochemical analysis. Thus, further studies using immunohistochemical analysis will be necessary to achieve a better understanding of ozone therapy on healing process in clean wounds.

## Conclusion

Ozone therapy applied topically and/or by bagging is not deleterious for the healing process of clean wound induced in rat’s skin. However, ozone bagging shows the best contribution to the healing process related to percentage of wound contraction and histological aspects of inflammatory infiltrate and tissue organization.

## Data Availability

All data were generated or analyzed in the current study.
